# Increased substantia nigra echogenicity correlated with visual hallucinations in Parkinson’s disease: a Chinese population-based study

**DOI:** 10.1007/s10072-019-04110-z

**Published:** 2019-11-22

**Authors:** Ting Li, Jing Shi, Bin Qin, Dongsheng Fan, Na Liu, Jingnian Ni, Tianqing Zhang, Hufang Zhou, Xiaoqing Xu, Mingqing Wei, Xuekai Zhang, Xiangzhu Wang, Jianping Liu, Yongyan Wang, Jinzhou Tian

**Affiliations:** 1 The Neurology Center, Dongzhimen Hospital, Beijing University of Chinese Medicine, Beijing, 100700 China; 2 Beijing Hospital, Beijing, 100730 China; 3 Peking University Third Hospital, Beijing, 100191 China; 4 Center for Evidence-Based Chinese Medicine, Beijing University of Chinese Medicine, Beijing, 100029 China; 5 Institute of Clinical Medicine, China Academy of Chinese Medical Sciences, Beijing, 100700 China

**Keywords:** Parkinson’s disease, Transcranial sonography, Visual hallucinations, Clinical features, Diagnosis

## Abstract

As a noninvasive technique, transcranial sonography (TCS) of substantia nigra (SN) has gradually showed its effectiveness not only in diagnosis but also in understanding clinical features of Parkinson’s Disease (PD). This study aimed to further evaluate TCS for clinical diagnosis of PD, and to explore the association between sonographic manifestations and visual hallucinations (VH). A total of 226 subjects including 141 PD patients and 85 controls were recruited. All participants received TCS. A series of rating scales to evaluate motor and non-motor symptoms were performed in PD patients. Results showed that 172 subjects were successfully assessed by TCS. The area of SN was greater in PD patients than that in controls (*P* < 0.001). As receiver-operating characteristic (ROC) curve analysis showed, the best cutoff value for the larger SN echogenicity size was 23.5 mm^2^ (sensitivity 70.3%, specificity 77.0%). Patients with VH had larger SN area (*P* = 0.019), as well as higher Non-Motor Symptoms Scale (NMSS) scores (*P* = 0.018). Moreover, binary logistic regression analysis indicated that SN hyperechogenicity (odds ratio = 4.227, *P* = 0.012) and NMSS scores (odds ratio = 0.027, *P* = 0.042) could be the independent predictors for VH. In conclusion, TCS can be used as an auxiliary diagnostic tool for Parkinson’s disease. Increased SN echogenicity is correlated with VH in Parkinson’s disease, possibly because the brain stem is involved in the mechanism in the onset of VH. Further studies are needed to confirm these findings.

## Introduction

Parkinson’s disease (PD) is one of the most common neurodegenerative diseases. Though dopamine transporter positron emission computed tomography (DAT-PET) is proved to be an effective diagnostic technique [1], it has not been widely used due to high expense and radio action. The diagnosis of PD mainly relies on clinical manifestations [2]. As a noninvasive technique, transcranial sonography (TCS) is potentially useful for the diagnosis of PD by showing the structural changes in substantia nigra (SN). Even though previous studies have proved that the specificity was 88.2–85% and the sensitivity was 84–94.9% in diagnostic accuracy of TCS in PD patients [3, 4], and the concordance rate between TCS patterns and PD diagnosis increased from 87 to 95% in a 4-year follow-up [5]. Still, the data based on Chinese population need to be supplemented.

It is still unclear whether the extent of SN hyperechogenicity correlates with only motor symptoms or other clinical status, more precise quantification of the damage is required in order to improve extensive and in-depth understanding in PD. There were studies exploring the correlation between SN echogenicity and clinical features, and found that patients with larger hyperechogenic SN area tended to have severer motor and non-motor symptoms [6–8]; however, visual hallucinations (VH), one of the most common psychotic symptoms, which was reported to affect 9.8 to 82.7% of PD population in different stages [9–11], in relation to SN, have not been reported before.

Our objective is to evaluate the validity of TCS for the diagnosis of PD in Chinese population, and to investigate the correlation of sonographic manifestations with clinical features, especially in VH.

## Subjects and methods

### Subjects

From May 2015 to March 2018, 141 participants with PD were recruited after giving their informed consent at the Department of Neurology, Dongzhimen Hospital. These 141 participants all met the criteria of the UK Parkinson’s Disease Society Brain Bank [2], and had enough audio-visual functions to complete motor and non-motor symptom evaluation test. Participants were excluded if they had possible dementia with Lewy bodies (DLB) according to 2005 DLB diagnostic criteria (DLBC-3) [12]. From May 2015 to January 2018, 85 volunteers without parkinsonism consented to participate as control subjects from the Department of Neurology, Dongzhimen Hospital and Poster recruitment. All volunteers were assessed by neurology specialists and were excluded if they had positive family history of PD or the possibility of parkinsonism.

This study had been approved by the ethics committee of Dongzhimen Hospital, the First Affiliated Hospital of Beijing University of Chinese Medicine.

### Transcranial sonography operation and diagnosis standard

**Fig. 1:**
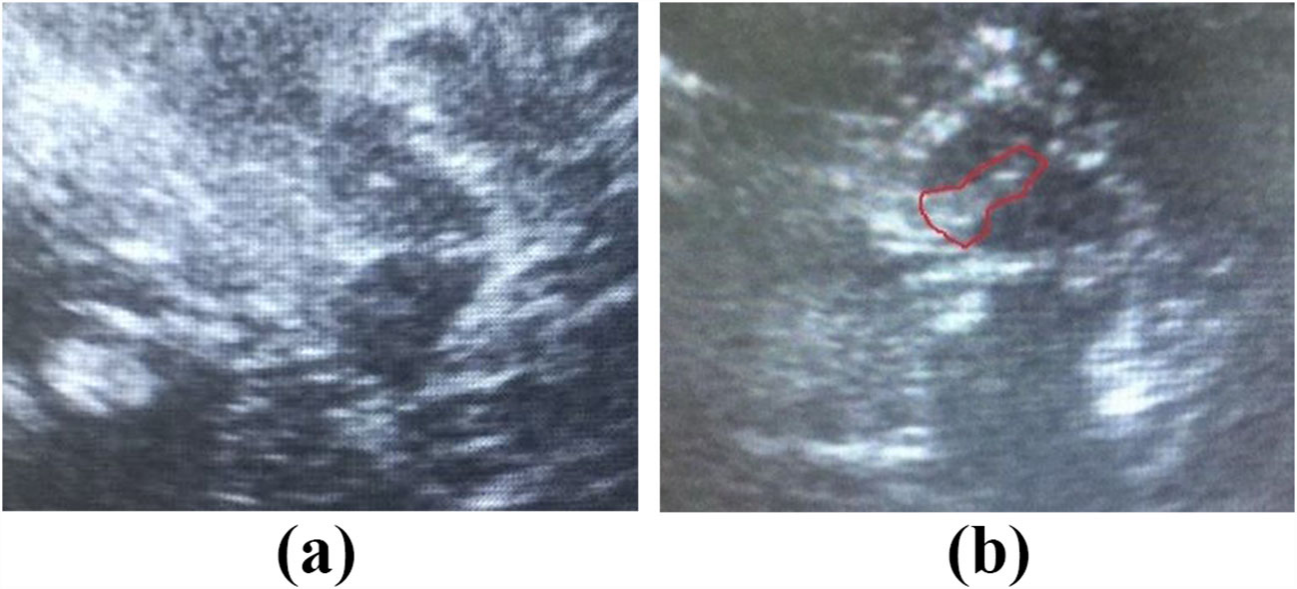
Sonographic images of the mesencephalic brainstem in a healthy control (**a**) and a patient with Parkinson’s disease (**b**). The butterfly-shaped mesencephalic brainstem was surrounded by the hyperechogenic basal cisterns. The patient with Parkinson’s disease exhibited hyperechogenic signals encircled by lines at both sides of SN, which were not seen in the control

Through the preauricular acoustic bone window, a qualified operator, who was blind to the clinical information of the subjects, examined the echogenicity of the SN using a 1.82 MHz sonographic device (ACUSON Anteres, SIEMENS) with a depth of 15–18 cm and a dynamic range of 26 dB. The SN was scanned through both temporal bone windows in the axial plane. After identifying the butterfly-shaped hypoechogenic midbrain surrounded by the hyperechogenic of basal cistern, the clearest image of the hyperechogenic signal in the SN region was stored (Fig. 1). The areas of SN hyperechogenicity in the midbrain were measured manually by the same operator.

The SN hyperechogenicity were obtained from right and left temporal windows. Some of the patients were measured from only one temporal window if it was impossible to obtain images from both sides. The larger SN echogenic area (SN_L_) was used to perform receiver-operating characteristic (ROC) curve analysis.

### Clinical features assessment

We recorded the clinical parameters including age, gender, disease duration, and symptoms of onset. To assess the disease severity, the PD patients received evaluation of the Unified Parkinson’s Disease Rating Scale (UPDRS) [13], and their Hoehn and Yahr Stage (H-Y stage) was graded during the 12-h medication “off” phase. Participants received a series of tests to evaluate their non-motor symptoms (NMS). The Non-Motor Symptoms Scale (NMSS) [14] was used to test the overall status. The PDSS [15], CSI [16], and PFS [17] were used to evaluate the participants’ sleep disorder, constipation, and fatigue symptom respectively. Mini-Mental State Examination (MMSE) [18] was used to evaluate the cognitive function. The degree of depression and anxiety was assessed by Hamilton Depression Scale (HAMD) [19] and Hamilton Anxiety Scale (HAMA) [20]. And the living quality was evaluated by PDQ-39 [21]. The presence of VH was defined according to item 13 from NMSS scale.

### Statistical analysis

The data were analyzed by using SPSS 22.0. The descriptive statistics were given as mean value ± standard deviation. The categorical statistics were recorded as count and percentage data. Descriptive statistics received normality test, and further variation analysis was performed by two-sample *t* test and Mann-Whitney *U* test. The statistical difference of categorical data was calculated by chi-square test. Correlations of the scale scores and SN_L_ were performed by Pearson correlation coefficients. Statistical significance was set at *P* < 0.05. The ROC curve analysis was applied to acquire the cutoff value to distinguish PD patients from normal controls. The classifier for ROC curve analysis was defined as The UK Brain Bank Criteria [2]. For each point in the curve, sensitivity and 1-specificity were shown for a certain cutoff value. And the best cutoff value was defined as where the sum of sensitivity and specificity was highest.

A binary logistic regression analysis was used to determine the most significant variables which were independently correlated with VH. As a dependent variable, the presence of VH was defined as a binary variable. Age, duration, SN_L_, UPDRS total scores, NMSS scores, and MMSE scores were included as covariates.

## Results

Among the 226 subjects, TCS was successfully performed in 172 subjects (76.10%, 111 PD patients, 72 men and 39 women, and 61 normal controls, 38 men and 23 women). Fifty-four subjects (23.90%) failed to acquire sonographic image due to poor penetration of ultrasound through both bony windows, and were excluded from further analysis.

### SN echogenicity in the participants

Table 1 shows the basic characteristics, scale evaluations, and SN echogenicity data among PD and control. There was no difference in gender and age between two groups. For PD patients, the mean SN hyperechogenic area was 21.61 ± 18.00 mm^2^ on the right side and 25.44 ± 20.24 mm^2^ on the left. For normal controls, the mean SN hyperechogenic area was 8.28 ± 14.58 mm^2^ on the right side and 12.44 ± 15.97 mm^2^ on the left, both were statistically smaller than PD group (*P* < 0.001, right side; *P* < 0.001, left side). The mean size of SN_L_ in PD group was 32.30 ± 18.42 mm^2^, which was significantly greater than the control group (14.61 ± 17.55 mm^2^, *P* < 0.001).

**Table 1:** Data of SN in the normal controls and PD patients

	PD patients (*N* = 111)	Controls (*N* = 61)	*P* value
Age (years)	66.35 ± 9.10	63.10 ± 11.44	0.059
Sex , male , *n* (%)	72 (64.86%)	38 (62.30%)	0.737
SN-right (mm^2^)	21.61 ± 18.00	8.28 ± 14.58	0.000*
SN-left (mm^2^)	25.44 ± 20.24	12.44 ± 15.97	0.000*
SN_L_ (mm^2^)	32.30 ± 18.42	14.61 ± 17.55	0.000*
UPDRS	34.33 ± 12.65	/	/
NMSS	49.73 ± 23.16	/	/
MMSE	27.28 ± 3.98	/	/
PDSS	115.21 ± 19.88	/	/
CSI	21.69 ± 11.67	/	/
PFS	47.58 ± 11.01	/	/
HAMA	11.26 ± 6.35	/	/
HAMD	8.68 ± 4.34	/	/
PDQ-39	32.52 ± 21.25	/	/

*SN* substantia nigra, *SN-right* right SN echogenic area, *SN-left* left SN echogenic area, *SN*_*L*_ the larger SN echogenic area, *UPDRS* Unified Parkinson’s Disease Rating Scale, *NMSS* The Non-Motor Symptoms Scale, *PDSS* The Parkinson’s Disease Sleep Scale, *CSI* The Constipation Severity Instrument, *PFS* Parkinson Fatigue Scale, *HAMA* Hamilton Anxiety Scale, *HAMD* Hamilton Depression Scale, *PDQ-39* 39 item Parkinson’s Disease Questionnaire

*P < 0.05

### Discriminative power of SN echogenicity for PD

**Fig. 2:**
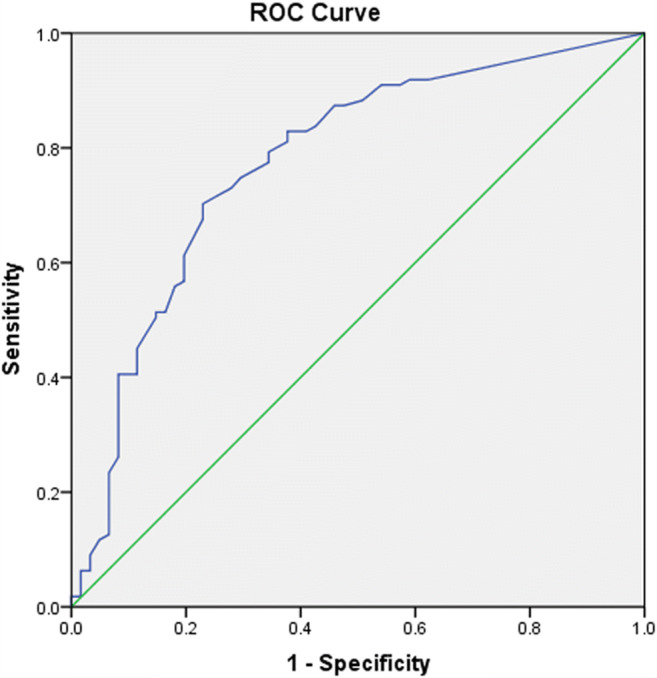
ROC curve for the differentiation of PD vs. control subjects. The larger SN echogenic areas (SN_L_) were taken for the plotting of the ROC curve in the entire cohort. Asterisk marks the point in the SNL curve for the cutoff of 23.5 mm^2^ (area under the curve = 0.775). The sensitivity value was 70.3%, and the specificity value was 77.0%

A ROC curve for the discrimination of PD patients and control subjects is shown in Fig 2. The larger SN echogenic areas (SN_L_) were taken to plot the ROC curve. The ideal diagnostic threshold should yield the highest sum of sensitivity and specificity. Therefore, the point situated in the top left corner of the curve would be the best diagnostic cutoff value. We marked the point in the SN_L_ curve for the cutoff value of 23.5 mm^2^. At this point, the sensitivity was 70.3%, and the specificity was 77.0%. The area under the curve was 0.775. Based on this cutoff value, 78 PD patients (70.27%) were classified SN_L_ ≥ 23.5 mm^2^; 14 control subjects (22.95%) were classified SN_L_ ≥ 23.5 mm^2^.

### Correlation between clinical features and SN echogenicity

We analyzed the correlation between age, H-Y stage, disease duration, UPDRS scores, UPDRS sub domain scores, NMSS scores, PDSS scores, CSI scores, PFS scores, HAMA scores, HAMD scores, MMSE scores, PDQ-39 scores, and SN_L_ with the Pearson correlation analysis. The UPDRS-II scores were significantly correlated with SN_L_ (*r* = 0.196, *P* = 0.039). Other scales did not show the correlation.

### Correlation of variables in PD with VH

In PD group, 18 (16.2%) of 111 patients had VH, including 4 possible dementia associated with Parkinson’s disease (PDD) and 14 cases with normal cognitive function [22]. We found that sonographic image and clinical features had special connection with VH; thus, we grouped the 111 patients according to with or without VH. As Table 2 shows, the mean age of VH group was much older than the non-VH group, and the difference had statistical significance (*P* = 0.008). The mean area of SN_L_ in PD patients with VH was larger than patients without VH in statistics (*P* = 0.018). The NMSS and UPDRS-II scores had statistical significance (*P* = 0.027, NMSS; *P* = 0.036, UPDRS-II) between VH group and non-VH group. The VH was not significantly associated with UPDRS total (UPDRS-T) and MMSE scores, although VH group had a higher level in UPDRS-T and a lower level in MMSE.

**Table 2:** Clinical data of PD patients grouped according to VH

	Total (*n* = 111)	With visual hallucination (*n* = 18)	Without visual hallucination *(n* = 93)	*P* value
Age (years)	66.35 ± 9.10	70.67 ± 8.85	65.52 ± 8.96	0.008*
Sex , male , *n* (%)	72 (64.86%)	15 (83.33%)	57 (61.29%)	0.073
SN-right	21.61 ± 18.00	31.06 ± 20.09	19.74 ± 17.06	0.028*
SN-left	25.44 ± 20.24	29.17 ± 23.65	24.72 ± 19.58	0.528
SN_L_	32.30 ± 18.42	41.67 ± 20.75	30.48 ± 17.48	0.018*
UPDRS-I	2.25 ± 1.72	2.94 ± 2.29	2.11 ± 1.57	0.201
UPDRS-II	12.41 ± 5.18	14.22 ± 5.56	12.06 ± 5.06	0.036*
UPDRS-III	18.20 ± 6.77	19.28 ± 6.62	17.99 ± 6.82	0.260
UPDRS-IV	1.47 ± 1.73	1.22 ± 1.48	1.52 ± 1.77	0.344
UPDRS-Total	34.33 ± 12.65	37.67 ± 12.87	33.69 ± 12.57	0.064
MMSE	27.28 ± 3.98	26.56 ± 3.52	27.42 ± 4.06	0.121
NMSS	49.73 ± 23.16	61.50 ± 28.48	47.42 ± 21.40	0.027*
Disease duration (years)	5.97 ± 5.57	6.17 ± 5.69	5.94 ± 5.58	0.939

*SN* substantia nigra, *SN-right* right SN echogenic area, *SN-left* left SN echogenic area, *SN*_*L*_ the larger SN echogenic area, *UPDRS* Unified Parkinson’s Disease Rating Scale, *MMSE* Mini-Mental State Examination, *NMSS* The Non-Motor Symptoms Scale

*P < 0.05

Further binary logistic regression analysis was used to identify the correlation between VH and other clinical features. Among age, duration, SN_L_, UPDRS total scores, NMSS scores and MMSE scores, SN_L_ and NMSS scores were the only two variables included in the final model. This demonstrated that there was a significant correlation between the VH and SN_L_ (odds ratio = 4.227, *P* = 0.012) and NMSS total scores (odds ratio = 0.027, *P* = 0.042).

## Discussion

This study explored the correlation between SN echogenicity and clinical characteristics in Chinese PD patients. We found that the SN echogenicity area in PD patients with VH was significantly higher than those without VH, which to our knowledge, had not been reported before.

As the results showed, 18 (16.2%) of 111 PD patients had VH, similar to another study in China (14.06%) [23]. Previous studies demonstrated that the impairment of visual input and central visual processing, as well as the impairment of brainstem regulation of the sleep-wake cycle may be the possible mechanisms [24, 25]. The exact pathogenesis of VH in PD patients is not clearly understood. Current studies concerning VH in PD and neuroimaging mainly used the technology of functional magnetic resonance imaging (fMRI), positron emission tomography (PET), and single photon emission computerized tomography (SPECT). Based on imaging studies, there was evidence which supported the hypothesis that abnormality and dysfunction in visual cortex and cholinergic structures such as the SN and pedunculopontine nucleus were to blame for VH [26]. However, the interaction between SN echogenicity and VH in PD was rarely investigated before.

Transcranial sonography can detect trace metal accumulation in deep brain structures with higher sensitivity than conventional MRI. In PD, particularly, the accumulation of iron has been suggested as an important substrate of extended SN echogenicity [27]. Berg reported close connections between SN echogenicity and elevated iron content of the SN in both animal and human studies [28, 29]. However, only a few cases of parkinsonism with VH have been reported with significant midbrain iron accumulation [30]. There was no direct evidence of correlation between iron accumulation in the SN and VH in PD. Another mechanism of enhanced SN echogenicity in PD might be microglial activation [31], which was demonstrated in midbrain specimens from postmortem PD patients [31, 32] and PD rat model [33]. A case report of PD with VH and delusions showed that neuronal loss with gliosis was noteworthy in the substantia nigra, locus ceruleus, dorsal vagal nucleus, nucleus basalis of Meynert, and intermediate lateral nuclei, and cholinergic projections from the nucleus basalis of Meynert could be responsible for generation of hallucinations and delusions [34]. However, there was also no direct evidence of correlation between microglial activation in the SN and VH in PD.

Among assessment of SN echogenicity, PD with VH had larger echogenic areas compared to those without. The correlation was further confirmed by binary logistic regression analysis. Together with these findings, we preliminarily inferred that the impairment of cholinergic structure in SN might contribute to VH in PD. Future studies may combine multiple imaging modalities to identify the main nerve damage in SN from iron accumulation, microglial activation, and other pathological processes.

Previous studies [35, 36] showed that VH in PD were associated with disease duration, dopamine agonist use, sleep quality, and cognition. In our study, there was significant difference between PD with VH and those without VH in age, NMSS, and UPDRS-II scores, while no statistic difference was found in disease duration, MMSE, and UPDRS-III scores. Moreover, there is no correlation between echogenicity and age, H-Y stage, NMSS scores, UPDRS-III, or disease duration. These results indicated that the correlation between VH and SN echogenicity was independent from NMSS scores, UPDRS-III scores, and disease duration. Considering there was no correlation between age and SN echogenicity in our study, similar conclusion was drew out in another Asian population-based study [8] (no difference in age between SN ≥ 18 mm^2^ group and SN < 18 mm^2^ group). We could initially speculate that the correlation between VH and SN echogenicity was also independent from age. However, PD with VH had higher UPDRS-II scores, and the UPDRS-II scores were associated with SN_L_. Therefore, we can not currently rule out the impact of UPDRS-II on the result that PD with VH had larger echogenic area.

There have been some studies concerning the relationship between SN echogenicity and clinical features of PD. Results showed that depression, urinary incontinence, UPDRS-II, SCOPA-AUT, and postural instability gait difficulty were related to SN hyperechogenicity [8, 37]. In our cohort, only UPDRS-II scores were associated with SN_L_, consistent with the result of Zhou’s study [8] based on Chinese patients. Yet, the underlying mechanism remains unclear. PD patients with VH had higher UPDRS-II scores might be a possible reason.

There has not been a well-accepted diagnostic value of hyperechogenic area to differentiate PD [8, 37]. Our study applied ROC curve analysis to investigate the cutoff value for SN+. The cutoff value in our study is higher compared with other researches [8, 38, 39]. To investigate the reason for this phenomenon, Table 3 demonstrates the clinical features of our study and 5 other studies [7, 8, 38–40]. The mean age was calculated using the information from all the participants, while the disease duration and H-Y stage were from only the PD patients. There seems to be a trend that as age and disease duration grow, the cutoff value also grows. Similar conclusion was carried out in a TCS hyperechogenicity area study [41] among healthy infants, children, as well as healthy adults. The result indicated an age-related increase of the hyperechogenic area of SN; thus, older age in our study might lead to an enlarged echogenic size level.

**Table 3:** Clinical characteristic comparison among different studies

	Cutoff value (mm^2^)	Mean age (years)	Disease duration (years)	H-Y stage
Our study	23.5	64.85 ± 11.23	5.97 ± 5.57	2.46 ± 0.70
Zhou H-Y [7]	18	60.77 ± 10.26	5.59 ± 4.21	2.02 ± 0.81
Berg D [28]	19	63	6	2
Kim J-Y [29]	20	56.9 ± 12.9	3.5 ± 3.2	2.06
Luo S-F [30]	20	58.7 ± 6.8	5.06 ± 3.11	-
Bartova P [6]	25	68.59 ± 10.37	6.8 ± 4.3	2

The TCS penetration rate was an important issue and might affect the study result to some extent, because some participants were excluded due to bad penetration of ultrasound through both bony windows. Among the 226 participants, 172 acquired adequate SN sonographic image; the insufficient penetration rate was 23.9% (21.3% in PD group, 28.2% in control group). In studies among Caucasian population, the insufficient penetration rate was in a level of 6.9–15.5% [7, 42, 43], while several Asian studies reported a higher temporal insufficiency rate varied from 20.5 to 30.2% [8, 39]. The Asian and Caucasian temporal bone structure diversity may cause the difference. Moreover, hyperostosis frontalis interna occurs as age grows, especially in female [44]. This might be another reason why the insufficient penetration rate was higher in our study.

In conclusion, enlarged SN hyperechogenic area significantly correlates with the presence of VH in a Chinese population with PD. This finding may provide evidence for brain stem involvement mechanism in the onset of VH. However, with a relatively small sample size of VH group, further studies are needed to confirm our findings.
